# Effect of Gaza war on the mental health of different generations in Egypt

**DOI:** 10.1186/s40359-025-03032-0

**Published:** 2025-07-03

**Authors:** Nadira Mansour Hassan, Rabab Ahmed Hammad, Amira Ahmed Abd El karem

**Affiliations:** Faculty of Medicine, Public Health and Community Medicine, Tanta University, Tanta City, Egypt

**Keywords:** War, Mental health, Depression, Anxiety, Stress, DASS-21, Students, Adults, Gaza

## Abstract

**Background:**

Exposure to unfavorable environmental circumstances including conflicts and wars increases people’s risk of experiencing mental health disturbances. Prevalence rates of anxiety, depression and stress disorders were two- to three-fold higher between people exposed to war or those interested in war news.

**Objectives:**

To explore prevalence of depression, anxiety and stress among Tanta University students and their parents representing different Egyptian generations, and to identify associated and predicting factors of severity and their effect on study or work performance.

**Methods:**

A cross-sectional study was conducted to collect data from undergraduate medical students at Tanta University and their parents by using random cluster sampling technique. The study included 100 medical students and 200 parents. A self-administrated questionnaire was used, which included sociodemographic data, DASS-21 (Depression, Anxiety Stress Scale) to measure levels of depression, anxiety and stress. Also, the questionnaire included a part to assess the effect of war on studying /or work performance. Multiple logistic regression was used to detect predictors of severe levels of depression, anxiety and stress. The value of *p* ≤ 0.05 was considered statistically significant. The data were analyzed using SPSS version 21.0.

**Results:**

Females constituted 79.0% of the student sample. All students and most fathers and mothers reported regularly following war news, with social media being the most frequently used source, especially among students. Symptoms of depression were reported by 97.0% of students, 77.0% of mothers, and 65.0% of fathers. Anxiety and stress were reported by approximately two-thirds of students, 51.0% of mothers, and 45.0% of fathers. Multiple logistic regression analysis identified frequent exposure to war news as a shared significant predictor of severe and extremely severe levels of depression (*p* = 0.004), anxiety, and stress (*p* < 0.001). Additionally, young age (*p* < 0.001) and female sex (*p* = 0.023) were significant predictors of depression, while urban residence was significantly associated with severe anxiety (*p* = 0.007). Students’ motivation to study and study hours were affected to some degree but among most parents, work performance was not affected (*p* < 0.001).

**Conclusion:**

The mental health of people in a country outside of war can also be significantly affected by war and its news. The students representing the younger generation were following war news frequently and were the worst affected generation. Thus, these findings highlight the importance of mental health screening and early intervention in populations not directly exposed to war but affected through media consumption, particularly across different generations.

## Introduction

Global rates of armed conflict have shown an alarming increase since 2008. Wars have devastating and long-term cumulative impacts on health. They directly result in death, injury, and destruction of infrastructure, and they cause widespread morbidity and mortality among both military personnel and civilians [1].

War-related morbidity includes a wide range of conditions—from disabling physical injuries to serious mental health disturbances. The World Health Organization (WHO) estimates that approximately 22.1% of individuals in conflict-affected populations develop mental health disorders, particularly depression, anxiety, and post-traumatic stress disorder (PTSD) [2, 3].

Mental health is a vital component of overall well-being. Poor mental health is associated with reduced productivity, a lower quality of life, and increased disability. In the modern era, millions of people are exposed to war-related content through media coverage. This exposure can result in psychological trauma—not only among direct participants or refugees, but also among those who are indirectly affected through repeated media exposure [4, 5].

Several studies have reported that citizens in countries such as Germany, Poland, the United Kingdom, and the United States experienced psychological disturbances—including anxiety, stress, and depression associated with the Russia-Ukraine war (RUW-22) [5, 6].

The long-standing Israeli Palestinian conflict has escalated repeatedly since the early twentieth century, creating a prolonged humanitarian crisis, particularly in the Gaza Strip. Gaza has been subjected to recurrent violence, resulting in a state of complex humanitarian emergency (WHO, 2022) [7, 8].

Since the onset of the Israel-Gaza war on October 7, 2023, global media attention has focused on the horrific consequences of the war on Gaza’s civilian population, including high rates of injury, death, destruction of infrastructure, and the struggle for physical survival. More than three-quarters (76.66%) of medical students in Gaza experienced severe to extremely severe stress [9]. The psychological impact of this war extends far beyond the borders of Israel and Gaza, affecting global audiences exposed to disturbing images and news coverage [10].

Given Egypt’s geographical proximity and shared border with the Gaza Strip, Egyptians were intensely engaged with war-related news during the early stages of the conflict. Therefore, it is important to assess the mental health impact of the 2023 Gaza war on different Egyptian generations. Medical students and their parents represent two distinct generational groups with different cultural perspectives and life experiences. Examining the mental health burden of the Gaza war on these groups may offer insights into the broader mental health status of the Egyptian population during such crises.

The overarching aim of this study was to assess the mental health burden of the Gaza war across two Egyptian generations (youth and adults).

### Objectives

The current study aims to:

1. Assess the level of depression, anxiety and stress among medical students in Tanta university and their parents during the first months of Gaza −23 war
2. Assess the relationship between frequency of following war news and mental health disorders.
3. Identify the associated and predicting factors of severity of DASS among studied groups
4. Identify the effect of war on academic and/or work performance.

## Methods

### Study design, duration, setting

The current study was a descriptive cross-sectional study. It was carried out during a period of four months from November 2023 throughout February 2024 at Faculty of Medicine, Tanta University which is the main university at middle delta in Egypt. Tanta City is the capital of Gharbia Governorate in the Middle Delta region of Egypt. It is 92 km north of Cairo.

### Study population, sample size and sampling techniques

The study participants were both sexes, Egyptian students from fifth year students at faculty of medicine and their parents. The sample size was dependent on the expected prevalence of 66.6% of mental problems according to Kasierska, et al. (2023) [11]. With total population of 2400 (800 students and 1600 parent), significance level 95%, precision 5% and by using epi‐info 7 software statistical package, the minimum required sample was 300 participants.

The sampling type was probability sampling in the form of cluster random sampling. The students in the 5^th^ year were already divided into rounds, nearly having the same numbers of students so, cluster sampling technique was used to get a representative random sample. The total number of students in the 5th year was 1070. However, the number of Egyptian students was 800 and the remaining were from other nationalities. The number of clinical rounds was 20 rounds (were considered as 20 clusters), each round contained around 50 students. All rounds were listed, each round had a number from 1 to 20, then three random numbers were chosen from random tables representing three chosen rounds to compensate the non-responsiveness, incomplete data and students from other nationalities. Each student was given three questionnaires, one for himself and two for his parents (one for his mother and another one for his father).

### Inclusion and exclusion criteria

- The inclusion criteria were:
  - Egyptian students of both sexes from Faculty of medicine, Tanta University and their parents after taking their voluntary consent to participate at the study.
- While the exclusion criteria were:
  - Students who are non-Egyptian, those who didn’t live with their parents and students who were incompletely filling out the questionnaires (either of themselves or their parents).

### Study tool

The pre-designed questionnaire included four parts; sociodemographic, frequency of following war news, DASS-21 scale Arabic version [12], and the fourth part was about academic/or work performance.

• The questionnaire includes 4 parts as follows:
  - Part I: Socio-demographic data including age, sex, residence, and qualification of parents.
  - Part II: Frequency of following the news. That part included 5 questions to estimate frequently used news outlets and news following frequency at the beginning of the war and after one month to identify the change at the rate of following the news.
  - Part III: the short version of the Depression, Anxiety, and Stress Scale (DASS 21, Arabic version)
  - Part IV: Academic/or work performance. That part included three questions. Each question had a three-point Likert scale as the following; not at all (1), to some extent (2), and to a large extent (3) to estimate the effect of following war news on the students’ academic study and work performance of parents. Each question was coded from 1 to 3. Total score was calculated ranging from 3 to 9, where higher scores reflect more affection.

### DASS-21 scale

This scale is the short version of DASS-42, the original manual. According to the DASS manual, emotional syndromes like depression and anxiety are intrinsically dimensional—they vary along a continuum of severity. Hence the selection of a single cutoff score to represent “clinical” severity is necessarily arbitrary. This is one of the main differences between DASS and categorical measures based on psychiatric diagnosis. Further, DASS authors developed a set of cut‐off scores for defining mild/moderate/severe/extremely severe scores for each DASS scale; as guided by the DASS manual. Psychometrically, DASS is quite different from diagnostic instruments in that it reflects the underlying continuity of severity of symptoms in the population; DASS scale scores are dimensional rather than categorical [13, 14].

On filling in the questionnaire, the participants were asked to indicate to what extent they had experienced each symptom during the past week (In contrast to the original manual, we asked about the first two months of the war”). DASS-21 included three subscales seven questions for each domain. The 21 items on the DASS were rated on a 4‐four‐point scale, where 0 corresponded to “did not apply to me at all” and 3 to “applies to me very much.”. in total DASS-21 consists of 21 questions which was designated for participants to specify their emotional level for each statement. Item scores were added together for each dimension and multiplied by two (as per DASS manual) to equal 42, to match the original scale**.** The minimum score is zero and the maximum score is 42. The final score of DASS can be categorized as in Table 1.

**Table 1:** Severity of depression, anxiety and stress [13]

Rating	Depression	Anxiety	Stress
Normal	0–9	0–7	0–14
Mild	10–13	8–9	15–18
Moderate	14–20	10–14	19–25
Severe	21–27	15–19	26–33
Extremely severe	28 +	20 +	34 +

DASS is a reliable tool to assess psychological distress in clinical and non-clinical populations [13, 15, 16]. Together, the subscales provide a broad-spectrum measure of psychological distress, indicating the severity and frequency of symptoms. DASS-21 requires less time to administer, and it is superior to the full-scale version [17].

### Validity of the study questionnaire

The questionnaire used in this study showed good validity, which was confirmed by three experts from the community and public health department and modifying the questionnaire according to their recommendations. Also, before data collection, a pilot study on 15 participants (5 students and 10 parents not included in the study) was used to test reliability of the modified questionnaire. Cronbach’s alpha was found to be 0.875 which indicates high internal consistency. The average time for fulfilling the questionnaire was 5–10 min.

### Data collection

The students were met during their study time at each round and data were collected at the beginning of the rounds to avoid examination times and stress inquired during these “hectic” and stressful periods of students’ academic lives. The objectives of the study were explained to the students who were informed that their participation was voluntary, and anonymity was assured. The participants were also told that the results of this research will be published. Each student was responsible for three questionnaires (one for him/herself which was fulfilled in the class and two for his parent). Questionnaires of students were obtained in class, and arrangements were made to bring back the complete questionnaires of parents.

### Statistics analysis of data

After collecting data, it was entered by excel sheet. After summation of the subscale scores for depression, anxiety and stress and their multiplication by 2 the investigators were recategorized the level of each domain as in Table 2.

**Table 2:** Severity of depression, anxiety and stress

Rating	Depression	Anxiety	Stress
Normal	0–9	0–7	0–14
Mild to moderate	10–20	8–14	15–25
Severe to Extremely severe	21–28 +	15–20 +	26–34 +

Distribution of participants in these categories was reported as frequencies and percentages. When variables met the normality condition (assessed by the Shapiro–Wilk test), parametric tests were used for analysis like ANOVA test which was used to compare means of more than one group and when not met the normality condition, non-parametric test (Kruskal–Wallis test) was used for analyses. Categorical data were presented as number and percentage and compared by chi square test for bivariate analysis, and it was replaced by Montecarlo exact when it was found inappropriate. Multiple logistic regression was used to detect predictors of severe levels of DASS. The value of *p* ≤ 0.05 was considered statistically significant. The data were analyzed using SPSS version 21.0

### Ethical consideration

Before starting the study, approval of the ethical committee at Tanta Faculty of medicine was obtained. After explaining research objectives and procedures, formal consent was obtained from all participants before filling in the questionnaires. The participants were free to choose whether they want to participate or not, they could withdraw at any time without any negative effects and they had a right to privacy and confidentiality. All authors have no potential conflict of interest.

## Results

The current study included 300 participants: one hundred medical students and their parents representing youth and young adult generations to assess depression, anxiety and stress levels.

Table 3 describes the sociodemographic characteristics of the study participants. The mean age of students was 22.31 ± 0.66 years old, corresponding to a mean age of 56.5 ± 5.0 among fathers and 49.5 ± 5.0 among mothers. The female students represented higher percentage (79.0%) than males (21.0%). More than half of the students were from rural areas (55.0%). Regarding the educational level of parents, university education represented the highest percentage (79.0%, 66.0% of fathers and mothers respectively) Table 4.

**Table 3:** Sociodemographic characters of the study participants

Variable	Students		Fathers		Mothers	
**Age/years**						
Mean ± SD	22.31 ± 0.66		55.8 ± 4.9		48.7 ± 4.3	
Range	21–24		48–65		42–58	
	*n* = 100	%	*n* = 100	%	*n* = 100	%
**Sex**						
Males	21	21.0	100	100.0	–	–
Females	79	79.0	–	–	100	100.0
**Residence**						
Urban	45	45.0				
Rural	55	55.0				
**Education level:**						
-Basic education (primary and preparatory)			13	13.0	20	20.0
-Technical education			8	8.0	14	14.0
- University education	100	100	79	79.0	66	66..0

**Table 4:** Follow up Gaza war news among the study participants (students and their parents)

Follow up of Gaza news status	Students (*n* = 100)		Fathers (*n* = 100)		Mothers (*n* = 100)		Total (*n* = 300)		χ^2^	P
	**n**	**%**	**n**	**%**	**n**	**%**	**n**	**%**		
**Do you follow news of Gaza war**										
Yes	100	100	99	99	97	97	296	98.7	**M.C**	**0.170**
No	0	0	1	1	3	3	4	1.3		
**Has your follow-up rate changed since the beginning of the war?**										
Decreased	57	57	41	41	51	51	149	49.7	**M.C**	**0.145**
Increased	30	30	34	34	29	29	93	31.0		
Did not change	13	13	25	25	20	20	58	19.3		
**Follow up during the first month of war**										
≤ every 2 h	43	43	27	27	22	22	92	30.7		
3 times a day	30	30	15	15	19	19	64	21.3		
Twice a day	10	10	16	16	22	22	48	16.0	**30.801**	0.001*
Once a day	10	10	28	28	24	24	62	20.7		
Few times a week	7	7	14	14	13	13	29	9.7		
**Follow up during the second month of war**										
≤ every 2 h	19	19	16	16	15	15	50	16.7		
3 times a day	18	18	15	15	14	14	47	15.7		
Twice a day	22	22	14	14	13	13	49	16.3		
Once a day	17	17	22	22	34	34	73	24.3	**21.714**	0.041*
A few times a week	20	20	21	21	10	10	51	17.0		
Once a week	4	4	12	12	14	14	29	9.7		
**Frequently used media**										
Social media networks	89	89	46	46	51	51	186	62.0	**MC**	0.001*
Television	9	9	51	51	46	46	106	35.3		
Digital news portals	2	2	3	3	3	3	8	2.7		

*MC* Monte-Carlo test

* Significant

Regarding the news, all students reported that they follow war news, vast majority of fathers and mothers (99.0%, 97.0% respectively). More than half of the students (57.0%) revealed that the rate of their following up to the news decreased, compared to 41.0% of fathers and 51.0% of mothers.

Follow up rate during the first month of the war; 43.0% of students checked the news every couple of hours or less and 30.0% of them checked it three times a day. Lower rates were observed among parents as only 27.0% of fathers checked the news every couple of hours versus 22.0% of mothers.

The most frequently used source of information was social media networks. It was reported by 89.0% of students, 46.0% of fathers and 51.0% of mothers. Television is still a main source of news for parents.

**Fig. 1:**
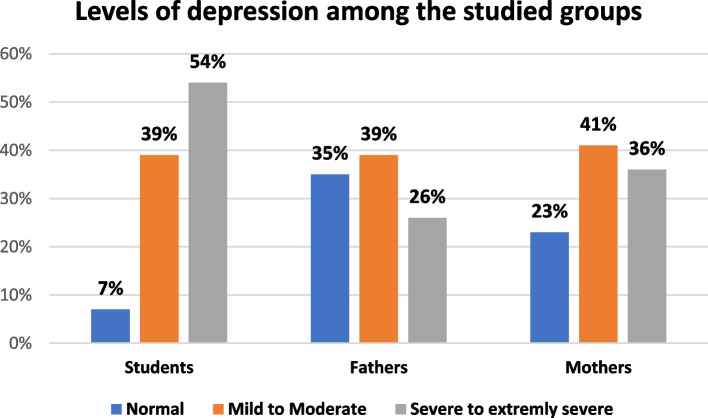
Depression levels among the study participants and their parents

**Fig. 2:**
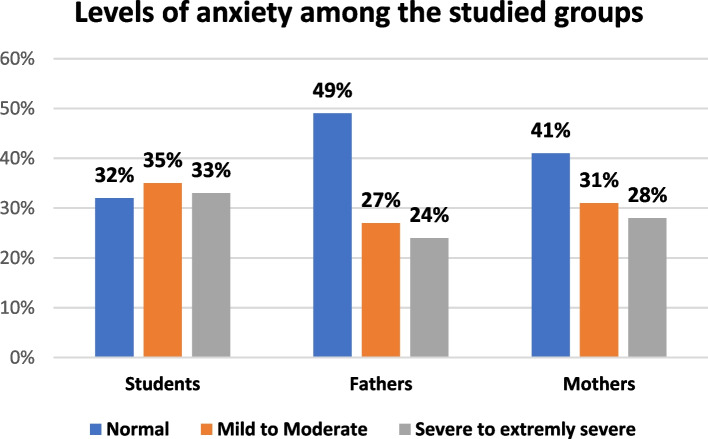
Anxiety levels among the study participants and their parents

Table 5 pointed to depression levels; severe or extremely severe depression symptoms were expressed by more than half of students (54.0%) followed by 36.0% of mothers and lastly 26.0% of fathers (Fig. 1). As regards anxiety levels, one third of students (33.0%) had severe to extremely severe anxiety followed by 28.0% of mothers and lastly 24.0% of fathers (Fig. 2).

**Table 5:** DASS—21 Scale levels among the study participants (students and their parents)

**DASS- 21 levels**	**Students**		**Fathers**		**Mothers**		**Significance test**
	**n**	**%**	**n**	**%**	**n**	**%**	
**1-Depression**							
-Normal	7	7.0	35	35.0	23	23.0	***X***^***2***^ = **28.696** *P *= < 0. 001*
- Mild to moderate	39	39.0	39	39.0	41	41.0	
-Severe to extremely Severe	54	54.0	26	26.0	36	36.0	
Median	**18**		**14**		**16**		**Kruskal–Wallis test** *P* = < 0. 001*
Interquartile range (IQR)	**10–26**		**9–22**		**10–24**		
**Post hoc Pairwise Comparisons**	Students-Mother 0.001*		Students-Fathers 0.001*		Mothers-Fathers 0.117		
**2- Anxiety**:							
-Normal	32	32.0	49	49	41	41.0	*X*^***2***^ = **6.025** ***P* = 0.197**
- Mild **to** Moderate	35	35	27	27	31	31.0	
-Severe to extremely severe	33	33	24	24	28	28.0	
Median	**10**		**8**		**10**		**Kruskal–Wallis test** ***P* = 0. 476**
Interquartile range (IQR)	**2–14**		**2–18**		**4–18**		
**3-Stress**							
-Normal	34	34	55	55	49	49.0	*X*^**2**^ = **9.436** *P* = 0.051*
- Mild **to** moderate	37	37	25	25	29	29.0	
-Severe to Extremely severe	29	29	20	20	22	22.0	
Median	**18**		**14**		**16**		**Kruskal–Wallis test** *P* = < 0. 001*
Interquartile range (IQR)	**14–26**		**8–20**		**14–24**		
**Post hoc Pairwise Comparisons**	Students-Mother 0.001*		Students-Fathers 0.001*		Mothers-Fathers 0.091		

*Significant

**Fig. 3:**
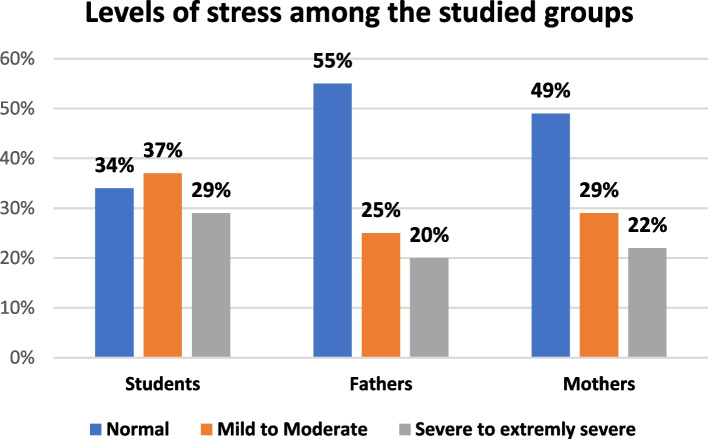
Stress levels among students and their parents

Pointing to stress levels, 29.0% of students had severe to extremely severe stress score followed by 22.0% of mothers and 20.0% of fathers (Fig. 3).

Table 6 showed univariant and multivariant analysis for the effect sociodemographic characteristics of all participants groups and frequency of war news checkup on depression, anxiety and stress disorders.

**Table 6:** Univariant and multivariant analysis for the effect of participants’ profile on depression, anxiety and stress levels

**Variables**	**Bivariant analysis**											
	**Depression**				**Anxiety**				**Stress**			
	Normal No (%)	Mild to Moderate No (%)	Severe ** No (%)	Sig. p	Normal No (%)	Mild to moderate No (%)	Severe ** No (%)	Sig. p	Normal No (%)	Mild to moderate %	Severe ** No (%)	Sig. p
**Age/years**												
≤ 24	7 (7)	39(39)	54(54)		32(32)	35(35)	33(33)		34(34)	37(37)	29(29)	
> 24	58(29)	80(40)	62 (31)	< 0.001*	90(45)	58(29)	52(26)	0.095	104(52)	54(27)	42(21)	0.013*
**Sex:**												
Male	36(29.8)	50(41.3)	35(28.9)	0.004*	55(45.5)	34(28.1)	32(26.4)	0.377	63(52.1)	31(25.6)	27(22.3)	0.196
Female	29(16.2)	69(38.5)	81(45.3)		67(37.4)	59(33.0)	53(29.6)		75(41.9)	60(33.5)	44(24.6)	
**Residence**												
Rural	29(17.6)	71(43.0)	65(39.4)	0.14	73(44.2)	58(35.2)	34(20.6)	0.004*	82(49.7)	48(29.1)	35(21.2)	0.331
Urban	35(26.6)	48(35.6)	51(37.8)		49(36.3)	35(25.9)	51(37.8)		56(41.5)	43(31.9)	36(26.7)	
**Qualification**												
Basic	8(24.2)	13(39.4)	12(36.4)	0.78	14(42.4)	11(33.3)	8(24.2)	0.432	15(45.5)	13(39.4)	5(15.2)	0.273
Technical	7(31.8)	8(36.4)	7(31.8)		13(59.1)	5(22.7)	4(18.2)		14(63.6)	4(18.2)	4(18.2)	
High	50(20.4)	98(40.0)	97(39.6)		95(38.8)	77(31.4)	73(29.8)		109(44.5)	74(30.2)	62(25.3)	
**News Checkup:**												
Frequently	23(15.8)	53(36.3	70(47.9)	0.003*	43(29.5)	48(32.9)	55(37.7)	< 0.001*	50(34.2)	50(34.2)	46(31.6)	< 0.001*
Infrequently	42(27.3)	66(42.9)	46(29.9)		79(51.3)	45(29.2)	30(19.5)		88(57.1)	41(26.6)	25(16.2)	
	**Multiple logistic regression** **Predictors of severe and extremely severe depression, anxiety and stress**											
	Depression				Anxiety				stress			
	Sig.(p)	Exp (B)	95% CI		Sig.(p)	Exp (B)	95% CI		Sig.(p)	Exp (B)	95% CI	
			L.B	U. B			L.B	U. B			L. B	U. B
**Age:** ≤ 24	< 0.001	5.383	2.198	13.188	0.282	1.425	0.747	2.719	0.101	1.729	0.898	2.329
**Sex:** female	0.023*	2.199	1.117	4.330	0.352	1.34	0.723	2.482	0.448	1.276	0.679	2.398
**Residence:** urban	0.099	0.596	0.299	1.011	0.007*	2.235	1.245	4.012	0.193	0.673	0.371	1.221
**Frequent checkup**	0.004*	2.644	1.354	5.161	< 0.001*	3.251	1.793	5.896	< 0.001	3.060	1.664	5.628

^*^ Significant,

^**^Severe: severe to extremely severe, Frequent checkup: ≤ *every couple of hours, 3 times a day, twice a day*

The univariant analysis showed statistically significant association between age and level of depression (*p* < 0.001) and stress (*p* = 0.013) where young age students (≤ 24 years) reported more severe and extremely severe manifestations of depression and stress (54% and 29% respectively) Vs parents (31%, and 21%) respectively. Also, sex showed a significant difference (*p* = 0.004) with severe and extremely severe depression level (45.3%) females Vs. 28.9% for males. There was significant difference between rural and urban participants regarding level of anxiety (*p* = 0.004) where urban participants were more associated with severe and extremely severe levels. Frequent checkup of war news was significantly associated with high levels of depression(*p* = 0.003), anxiety and stress (*p* < 0.001).

Multiple logistic regression analysis showed that the shared predictor that pointed to severe and extremely severe level of depression (*p* = 0.004), Anxiety and stress (*p* < 0.001) was frequently checkup of war news. On the other hand, young age (*p* < 0.001) and female sex (*p* = 0.023) were significant predictors for depression and urban residence was a significant predictor for severe and extremely severe level of anxiety.

Table 7 revealed that war events had significant effect on study/or work performance among the study participants (*p* = 0.04*). Post hoc pairwise showed that the effect on students was statistically significant different from mothers (*p* < 001) and fathers (*p* < 001). Motivation for study, hours of study and grades were affected to some degree for most students (48%, 50% and 56% respectively) corresponding to only about one third of fathers and mothers. Also, most fathers (61%) and mothers (53%) their work performance was not affected at all.

**Table 7:** Effect of war events on study/or work performance among the study participants (students and their parents)

Variable	Students (*n* = 100)		Fathers (*n* = 100)		Mothers (*n* = 100)		Total (*n* = 300)		Sig. test	*p*-value
	n	%	n	%	n	%	n	%		
Motivation to study/or work decreases due to the events of the war:										
-To a great degree	30	30.0	16	16.0	18	18.0	64	21.3	χ^2^ 25.669	< 0.001*
-To some degree	48	48.0	31	31.0	30	30.0	109	36.3		
-Not at all	22	22.0	53	53.0	52	52.0	127	42.3		
Number of hours you study/or work affected:										
-To a great degree	21	21.0	15	15.0	14	14.0	50	16.7	χ^2^ 23.132	< 0.001*
-To some degree	50	50.0	24	24.0	33	33.0	107	35.7		
-Not at all	29	29.0	61	61.0	53	53.0	143	47.7		
Performance/or grades decrease due to the events of the war:										
-To a great degree	14	14.0	8	8.0	11	11.0	33	11.0	χ^2^ 20.965	< 0.001*
-To some degree	56	56.0	31	31.0	36	36.0	123	41.0		
-Not at all	30	30.0	61	61.0	53	53.0	144	48.0		
Total score of effect of war on the study participants										
Mean ± SD	7.4 ± 1.7		6.7 ± 2.1		6.9 ± 2.0		7.0 ± 2.0		*F* = 3.250	0.040*
Post hoc Pairwise Comparisons	Students-Mother 0.001*		Students-Fathers 0.001*		Mothers-Fathers 0.464					

* Significant

## Discussion

The present study was conducted at the Faculty of Medicine, Tanta University, and involved fifth-year medical students and their parents, representing two distinct generations of the Egyptian population. The primary aim was to explore the mental health burden of the Gaza War-2023 on youth and adults in Egypt, specifically focusing on depression, anxiety, and stress and identify associated and predicting factors of severity and their effect on study/or work performance.

To the best of our knowledge, this is the first study that dealing with mental health burden among population of different generations in Egypt (a country that has share borders with the conflict zone). At the time of carrying out of this study, Gaza war-23 was about to end its second month. The key findings of this study could be summarized as follows; Firstly, during the first months of Gaza war −23, there was great mental distress among the studied population with significant difference between generations. Second, all students and most parents (fathers and mothers) reported regularly following war news. Third, females whether youth or adults were more susceptible to mental distress when exposed to stressed environment. Fourth, social media network was the favourite source of news for youth and television still constitutes important for adults. Fifth, the shared predictor that pointed to severe and extremely severe level of depression, Anxiety and stress was frequently checkup of war news. On the other hand, young age and female sex were significant predictors for depression and urban residence was a significant predictor for severe and extremely severe level of anxiety. Sixth, war events had a noticeable effect on youth than adults manifested by the significant differences in desire to study/or work, hours of study/or work and total score of effect of war events.

Wars are public health emergencies that affect human lives at multiple levels. In addition to immediate casualties and physical suffering, wars leave survivors with long-term psychological consequences [18]. The Gaza War-2023 represents a massive humanitarian crisis following the Russia-Ukraine War (RUW-22), and the devastation is traumatic not only for those directly involved but also for individuals in neighboring countries and beyond. However, unfortunately, no studies have examined the effect of war on civilians of different generations, in Arabic countries.

Our findings indicated that female students constituted a higher proportion (79%) of the student participants, largely due to the sex distribution within the target population. A similar pattern was observed among residents of Jordon at the study that was published by Al-Ajlouny et.al. (2025) to explore the psychological effects of Gaza war where females were constituting 77.3% of the studied sample [10]. Also, Moya-Salazar et al. (2025) in Peru, revealed that more than one half of Peruvian university participants were females [19].

Based on the DASS-21 assessment, at the current study, over half of the students (54%) experienced severe to extremely severe depression manifestations and this was higher than that reported among university students (40%) in the Czech Republic following RUW-22 [18]. Similarly, the levels of depression observed among parents in our study were greater than those reported in general populations in Poland and Taiwan—countries indirectly affected by RUW-22 [20]. This discrepancy may be attributed to the emotional nature of the Egyptian population and the close cultural and historical ties between Egyptians and Palestinians. Supporting this, Hendawy et al. (2024) identified that being Egyptian as a univariate predictor of elevated general distress (*p* = 0.014) [21].

Our findings indicated that female students constituted a higher proportion of the student participants, largely due to the sex distribution within the target population. A similar pattern was observed by Moya-Salazar et al. (2025) in Peru, where most university student participants were females [22].

Multivariate analyses during RUW-22 in Poland, Ukraine, and Taiwan found that females were significantly more likely to experience higher DASS-21 scores [20]. Our study reinforces the notion that females—both young and adult—are more vulnerable to psychological distress in times of crisis. Numerous previous studies have also highlighted sex-based differences in responses to stressful environments [21–25].

The risk of psychological distress among those indirectly affected by war is closely linked to exposure to violent content and distressing images circulated via social media [5]. Nearly all participants in our study followed news of the Gaza War, with notable differences in frequency and sources of information. Approximately half of the students checked for updates every few hours or more frequently, whereas only about one-fifth of parents did so. Most students (89%) relied primarily on social media for war news, while parent accessed both social media and television. This discrepancy in media consumption may explain the higher DASS-21 scores observed among students, aligning with previous research indicating Generation Z’s reliance on social media for news [26, 27].

Our findings support those of Chudzicka—Czupała et al. (2023), who reported that people living outside war zones can experience mental health problems through exposure to distressing war content in the media [20]. The nearly constant coverage of the Gaza War on TV and the internet made traumatic images widely accessible. Prior studies [18, 20, 21, 28–30] confirmed that frequent exposure to such content intensifies psychological distress. Our results were in consistent with these studies and revealed that the shared predictor that pointed Russia–Ukraine War to severe and extremely severe level of depression, anxiety and distress was frequently checkup of war news. Recently, Hendawy et al. (2024) found that such exposure was a significant predictor (OR 3.26, 95% CI 1.78 to 5.99, *p* < 0.001) of both general and significant psychological distress among Egyptian and Jordian populations [21].

Notably, the frequency of following Gaza war news declined over time, especially among students (57%) and this was also observed by Kasierska et al. (2023) who analyzed risk factors for depression and anxiety in Polish population in the context of the War in Ukraine at first, second and after six months of the conflict [11]. De Bruin et al. (2021) attributed such behavioral shifts to “news fatigue,” caused by information overload and emotional exhaustion [31]. This decrease may also represent a coping strategy. Xu et al. (2022) reported that self-distraction was an effective coping mechanism that helped reduce anxiety, depression, and insomnia in Ukrainian participants [28].

The present study pointed to urban residence as a significant predictor for severe levels of anxiety among participants (*p* = 0.007) and this agreed with the findings of Danek et al. (2023).who observed that individuals living in urban areas were more likely to report worsening anxiety than those living in rural areas during the COVID‐19 pandemic and considered that as a reflection to urban and rural population differences in the perception of crises events [32].

The current study revealed that war-related effect of mental distress—manifested as depression, anxiety, and stress—varied between students and their parents. Zhang et al. (2024) identified a strong association between psychological distress and academic performance, with mental health emerging as a predictor of academic outcomes [33]. Similarly, Ntoiti et al. (2024) concluded that high levels of psychological distress negatively impact academic achievement among undergraduate students [34].

On the other hand, Elotla et al. (2021) in Egypt [35] and de Oliveira et al. (2023) [36] confirmed that poor mental health was associated with reduced productivity. These findings aligned with our results, which showed that students’ motivation to study, study hours, and academic performance were negatively affected more than work performance among parents, who may have had other life responsibilities to manage. The overall effect of war-related psychological distress on academic/or work performance was notably higher among students than among parents, highlighting generational differences in vulnerability and coping mechanisms.

### Limitations of the study

As the study was a cross-sectional one it misses the causality relationships. Also, it included a single academic year from a single medical university with a lack of comparability in addition to the smaller number of students enrolled in the study. Moreover, the females constituted higher proportion in the study which may not reflect the real burden of the problem inside the community. Recalling bias may be present as the participants were asked to describe their condition two months ago. The study’s reliance on self-reported data, which could result in errors because of recall bias or forgetfulness, is another drawback. Multicenter research and community-based studies on the topic can offer additional insights into mental burden of war on youth and adult population who represent the core population of Egypt community.

## Conclusion

The present study showed that the mental health of people in a country outside of war can also be significantly affected by war and its news. The students representing the younger generation were following war news frequently and were the worst affected generation. This study pointed out that in countries outside wars, screening for different mental health problems, their determinants and predictors among different generations is crucial for identification of people whose condition requires specialized interventions. Future studies are recommended to explore the long-term effect of this war on different generations and on general Egyptian population.

## Data Availability

Data is provided within the manuscript or supplementary information files and any other information could be obtained from the corresponding author on reasonable request.
